# α-Pyrrolidinohexanophenone (α-PHP) and α-Pyrrolidinoisohexanophenone (α-PiHP): A Review

**DOI:** 10.3390/life14040429

**Published:** 2024-03-22

**Authors:** Pedro Dinis, João Franco, Cláudia Margalho

**Affiliations:** Laboratory of Forensic Chemistry and Toxicology, National Institute of Legal Medicine and Forensic Sciences, I.P.—Centre Branch, Pólo das Ciências da Saúde (Pólo III)—Azinhaga de Santa Comba, 3000-548 Coimbra, Portugal; pedro.a.dinis@inmlcf.mj.pt (P.D.); j.miguel.franco@inmlcf.mj.pt (J.F.)

**Keywords:** α-PHP, α-PiHP, NPS, forensic toxicology

## Abstract

New Psychoactive Substances are currently a serious and growing problem affecting public health worldwide. By 2022, 1184 of these substances had been identified over a period of 16 years. Within these, α-pyrrolidinohexanophenone (α-PHP) and α-pyrrolidinoisohexanophenone (α-PiHP) have emerged, two synthetic cathinones from the pyrovalerone derivates subgroup that are positional isomers of each other. Alpha-PHP appeared on the Japanese illicit drug market in 2014 and, two years later, α-PiHP was identified for the first time in China. They were placed in schedule II on the list of Psychotropic Substances under International Control in 2020 and in March 2023, respectively. Both cathinones have no therapeutic potential for medical use and therefore are abused for recreational habits, which can lead to fatalities. The most frequent adverse effects reported are cardiac, psychiatric, and neurologic, and fatal intoxications have already been described. In Portugal, their consumption and consequent seizures are more prevalent on the archipelagos, which has been aggravating the health situation. In conclusion, these types of substances are a challenge for forensic toxicology since they are easily synthesized, modified, and placed on the market. Therefore, more studies to develop analytical methods to detect them and more comprehensive legislation should be applied. Thus, this review aimed to address the legislative, physicochemical, toxicological, and analytical aspects of both substances.

## 1. Introduction

New Psychoactive Substances (NPSs) are characterized as substances in a pure or prepared form that are not under the 1961 and 1971 European Union Conventions and can jeopardize public health. They are rapidly emerging and spreading through the market, triggering social and public health risks [1]. The global market growth of NPSs is a concern for justice entities, since the compounds’ modifications contribute to hindering their identification and detection. It also has an impact on human health due to NPSs’ unknown composition and purity, which is a consequence of homemade production in illegal laboratories [2]. Thus, NPSs are gaining popularity on account of the lack of legislation for these substances and their large-scale and easy accessibility through the online market [3]. In 2022, worldwide, 1184 NPSs were identified (cumulative number of the previous 16 years), although they presented a lower consumption rate than traditional drugs [4]. The European Monitoring Centre for Drugs and Drug Addiction (EMCDDA) was monitoring about 930 NPSs at the end of 2022, with synthetic cathinones representing the second largest group of substances monitored until 2021 [5].

**Figure 1:**
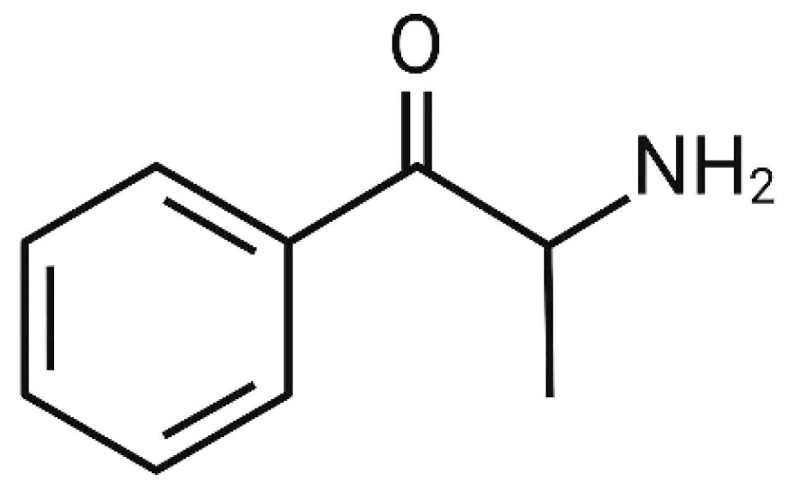
Chemical formula of cathinone.

Synthetic cathinones are a group of psychostimulant designer drugs that are included in the NPS category. They are usually labelled with “not for human consumption”, “jewellery cleaner”, and “phone screen cleaner” and sold as “bath salts” and “plant food” in order to convey a false idea of security and legality. The aim of their development is not only to provide similar effects to conventional drugs of abuse, such as amphetamines, 3,4-methylenedioxymethamphetamine (MDMA), and cocaine, but also to be less expensive and easily obtained, triggering their replacement. The synthetic cathinones group derives from the natural stimulant ingredient produced by cathinone (Figure 1), which is found in the leaves of the khat plant (*Catha edulis*) [6,7,8,9,10]. Cathinone is an alkaloid with a similar structure and effects to amphetamine, and its most common routes of administration are nasally and orally [6,9]. However, when harvested, the fresh leaves of the khat plant can be chewed, as occurs in Yemen and East African countries as a social habit [11]. The administration of synthetic cathinones can cause euphoria and increased alertness, but their abuse can lead to serious cardiac, psychiatric, and neurologic effects [8,9]. When consumed in higher doses, they can provoke delirium and hallucinations, hyperthermia, tachycardia, and hypertension [12].

Novel derivative compounds have emerged in recent years from the synthetic cathinones group, including α-pyrrolidinohexanophenone (α-PHP) and α-pyrrolidinoisohexanophenone (α-PiHP). Both belong to the pyrovalerone derivatives subgroup (α-pyrrolidinophenones), which is chemically different from other cathinones and is characterized by a pyrrolidine ring and an α-carbon side chain [13]. Alpha-PHP was internationally scheduled at the end of 2020 and became part of Portugal’s legislation in May of 2021 [14,15]. According to national news organizations, this synthetic drug has widely spread in the archipelago of Madeira, causing severe health problems for the youth generation [16,17]. In contrast, α-PiHP is an isomer of α-PHP and a recently internationally controlled substance, while in Portugal, is currently not controlled [18].

Alpha-PHP is a synthetic stimulant drug from the cathinones group which was developed in the 1960s [19]. In April of 1967, the United States Patent Office published a patent document related to α-pyrrolidine ketones and synthesis methods of their novel substances, including the α-PHP compound. However, this patent expired in 1984, triggering its illicit manufacturing [20]. It was identified in material seized from the Japanese illicit drug market in 2014, and was implicated in drug-related intoxication deaths a few years later [21,22]. On the other hand, α-PiHP is an α-PHP chain isomer and was first reported internationally in 2016 by Liu et al., who identified and characterized it analytically along with eight synthetic cathinones in seized materials in China [23]. In December of the same year, α-PiHP was formally submitted to the Early Warning System of EMCDDA, and in 2019, its concentration in biological specimens was determined for the first time in an intoxication case [24]. Since 2016, α-PiHP has been reported to the United Nations Office on Drugs and Crime by 30 countries and territories worldwide, including Europe, Asia, North America, and Oceania [18].

This review intends to compile the most recent information available about α-PHP and α-PiHP regarding their current situation worldwide, chemical properties, and toxicological aspects.

## 2. Current Situation

Nowadays, the number of NPSs on the global market is increasing, after several years of stabilization, due to traffickers who continue to sustain their production. Consequently, the prevalence of their use is higher, especially in younger age groups [4,5].

Both α-PHP and α-PiHP have no medical use in therapeutics, and they are abused as novel designer drugs for recreational habits, leading to fatalities [19,25]. Klavž et al. reported a suicide attempt with psychoactive substances, including α-PHP. The patient was somnolent and unresponsive to painful stimuli and presented dilated pupils, tachycardia, and pronounced dehydration [26]. Beck et al. presented a study of 114 confirmed intoxication cases with pyrovalerone compounds. In 93% of the cases, the authors verified the combined use of different NPSs and of NPSs with classical drugs, while in 7% of cases, a single pyrovalerone drug had been abused. In one of these cases, α-PHP was detected, and the patient had dilated pupils, paraesthesia, hypertension, and respiratory alkalosis [27]. Vignali et al. identified and quantified α-PHP in a deceased person with a history of drug use. The analysis was performed on biological specimens such as blood, urine, bile, gastric contents, liver, kidney, spleen, lung, brain, heart, and hair [28]. Adamowicz et al. carried out an investigation into a suspected foetal intoxication death due to the maternal consumption of α-PHP and MDPHP. Foetus death was a result of intrauterine asphyxia, and its α-PHP blood concentration was much higher compared to the maternal values [29]. Lagoutte-Resoni et al. reported two case histories of acute NPS intoxication. In one case, the patient was admitted to the emergency unit after jumping from the first floor of a building with signs of agitation and disorientation, logorrhoea, and hallucinations. Blood, urine, and hair were analysed and confirmed the presence of α-PHP [30]. Pieprzyca et al. analysed drug addiction cases, of which 26 were fatal and 2 non-fatal. Regardless of all the drugs identified, α-PHP was detected in ten cases with a blood concentration range between 10 and 153 ng/mL, and a urine concentration from 10 to 2520 ng/mL. All these cases were based on polysubstance intoxication leading to the demise of nine subjects [31]. Grapp et al. researched α-PHP intake in 29 forensic and clinical cases, of which two were newborns. Serum specimens were used for α-PHP quantitation, and in some cases, since it was absent, meconium in newborn cases, blood from the femoral vein, and urine were analysed. The predominant symptoms in the reported cases were aggressive behaviour and resistance to the authorities, coupled with a delayed physical response and drowsiness [32]. Adamowicz et al. reported a case of α-PiHP intoxication and achieved its determination in biological specimens. Before death, the person was agitated, running, wheezing, hallucinating, and talking to himself [24]. Zawadzki et al. reported an intoxicated couple found at home, where the man was unconscious, and the woman was lifeless. The man was unresponsive to light, with highly constricted pupils, and had muscle tremors, and the screening test was positive for amphetamines. Alpha-PiHP was identified in both, but in higher concentrations in the man [33]. Wachholz et al. carried out an investigation of a man’s fatal poisoning with α-PiHP. As reported before his demise, the man was agitated and acting strange, and afterwards, he lost consciousness [34].

Concerning global regulations, substances, certain chemicals, and drugs are classified into five schedules depending on their medical use and their dependence and abuse potential [35]. Thus, in 2020, the International Narcotics Control Board (INCB) listed α-PHP as a schedule II drug on the list of Psychotropic Substances under International Control following the Convention on Psychotropic Substances of 1971 [36,37]. In addition, in 2022, the Drug Enforcement Administration (DEA), part of the Department of Justice of the United States of America (USA), updated the schedules of controlled substances, placing α-PHP in schedule I [38]. Furthermore, α-PHP is in class B in the United Kingdom, and it is illegal in China, Sweden, Poland, and Italy [19]. In contrast, since it was first time reported in 2016, α-PiHP was placed in schedule II in March 2023 through the Convention of Psychotropic Substances of 1971 by the INCB. Currently, this is the only legislation applicable to α-PiHP worldwide [36].

In Portugal, NPSs have been emerging and gaining relevance since 2005, although their designation was only implemented in 2012. The first national smartshop/headshop dedicated to the legal commercialization of these substances opened in 2007, which increased the consumption among youth [10,39]. The following six years showed exponential growth, with around 63 smartshops opened by 2013, which can be explained by consumer demand triggered by curiosity, and by the emergence of new synthetic compounds [39,40]. At the end of 2012, the first survey of severe intoxication cases was carried out in hospital emergency departments by the Directorate-General for Health of Portugal (DGS) [41]. Furthermore, based on alarming drug-related deaths and hospitalizations in 2012, the autonomous region of Madeira was a pioneer in the fight against NPSs, approving on October 25th Regional Legislative Decree number 28/2012/M. This decree addressed the ban on the possession, production, sale, and distribution of NPSs, contributing to the closure of smartshops in the region [42,43,44]. One year later, and considering the dangerousness of NPSs, Decree Law number 54/2013 was published at a national level, together with Ministry of Health Ordinance number 154/2013, both on 17 April. This decree defined a legal regime for prevention and protection against the advertising and trade of NPSs, listed in the ordinance, as well as sanctions for those who would infringe it [45,46]. As a result of the measures taken, all the smartshops were closed and the consumption of NPSs decreased abruptly. Nevertheless, the internet and social media markets have become increasingly powerful when it comes to obtaining these substances [10,39,40,45,46,47]. The 2012 decree of Madeira has been updated twice through Regional Legislative Decree number 7/2017/M of 8 March and number 13/2023/M of 14 March, always with the aim of protecting public health and of punishing those who are involved [48,49].

Currently, the autonomous regions of Madeira and Azores have a higher prevalence of NPSs than the mainland of Portugal, which may be motivated by the decreased accessibility and increased cost of traditional drugs (heroin, cocaine, and others) [40,50,51,52]. Regarding α-PHP, its first appearance was in 2021 and it was included in the legal regime applicable to the trafficking and consumption of narcotic drugs and psychotropic substances (Decree-Law number 15/93 of 22 January1993) by Law number 25/2021 of 11 May [50,53,54]. However, more and more seizures of α-PHP, commonly known as “bloom”, continue to be reported today, both for consumption and for trafficking, and most of them involve young people, which has led to more fatal cases and hospitalizations [51,55,56,57,58]. In contrast, α-PiHP was first detected in Portugal in 2023, more specifically, firstly in Azores and secondly in Madeira [59]. This substance is not considered in the national legislation, and not much is known about it due to its recent appearance.

The problems associated with the consumption of α-PHP and -PiHP are the increase in reported fatal and non-fatal intoxications and consumers being unaware of the product they are consuming in most cases. Thus, there is a need to implement more efficient methods to detect NPSs, to modernize the regulations on drugs, and, more importantly, to raise awareness within the population of their related risks and implications.

## 3. α-Pyrrolidinohexanophenone (α-PHP)

### 3.1. Identification

Alpha-PHP or PV-7, as mentioned above, is a synthetic stimulant of the cathinones class, which is part of the pyrovalerone derivatives subgroup [19,60]. Its physical appearance can vary from transparent to whitish or yellowish crystals, and from beige to brownish powder, granulated or ungranulated. In fact, the brownish colour of the powder form can be explained either by synthetic impurities or by the powder being a freebase and the crystals constituting a HCl salt [19,61]. According to the World Health Organization (WHO), α-PHP has a flavour that tends towards bitter, being sold on the streets under the same names as synthetic cathinones in general, except in Portugal, where is known as “bloom”, as previously mentioned [19,57].

### 3.2. Chemistry

**Figure 2:**
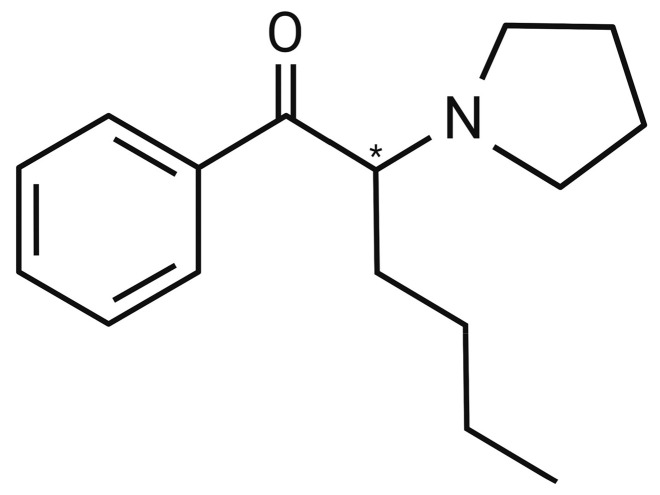
Chemical formula of α-pyrrolidinohexanophenone (α-PHP).

Regarding its chemical properties, α-PHP’s IUPAC name is 1-Phenyl-2-(pyrrolidine-1-yl)hexan-1-one and it has a molecular weight of 245.36 g/mol [62,63]. Its structure (C_16_H_23_NO) is composed of a hexanal bonded to a phenyl ring at position number 1, and to the nitrogen of a pyrrolidine ring at the position 2, and it has a chiral centre (*) in the α-carbon of the side chain, as illustrated in Figure 2 [19,64]. Moreover, the melting and boiling points are 108.90 °C and 339.06 °C, respectively, and the water solubility (25 °C) is established at 39.83 mg/L [19].

**Figure 3:**
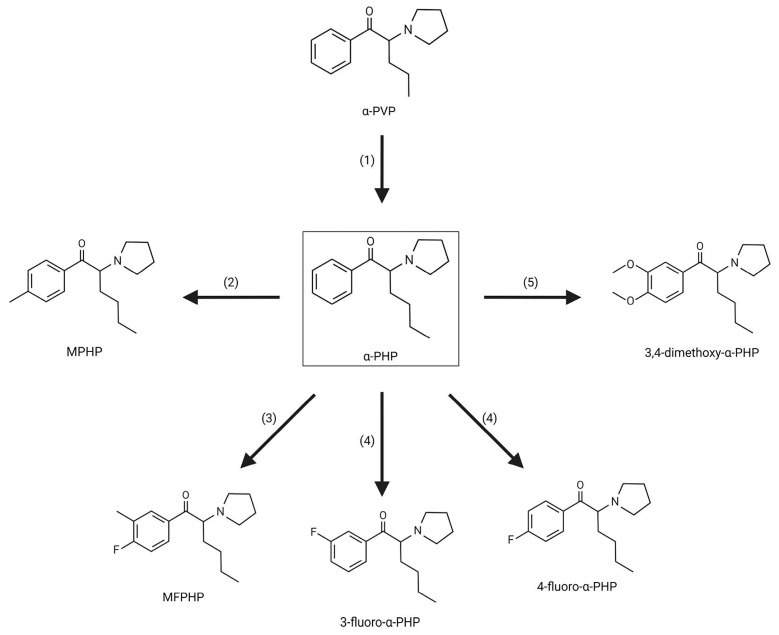
α-PHP analogues: (1) modification of alkyl side chain; (2) substitution of position 4 of the phenyl ring with a methyl group; (3) association of a halogen atom and a methyl group in phenyl ring; (4) substitution of positions 3 or 4 of the phenyl ring with a halogen atom; (5) substitution of positions 3 and 4 of the phenyl ring with a methoxy group.

Within the pyrovalerone derivatives subgroup, the most prominent compound is 3,4-methylenedioxypyrovalerone (3,4-MDPV), since after its appearance on the market, many others began to emerge [13]. In fact, through the methylenedioxy deletion of 3,4-MDPV is born α-pyrrolidinopentiophenone (α-PVP). From the latter it is possible to synthesize α-pyrrolidinobutiophenone (α-PBP) and α-pyrrolidinopropiophenone (α-PPP) by shrinking the alkyl side chain, and α-pyrrolidinohexanophenone (α-PHP), α-pyrrolidinoheptanophenone (α-PHPP), and α-pyrrolidinooctanophenone (α-POP) by extending it. Furthermore, α-PHP can be processed through the substitution of positions 3 and/or 4 of the phenyl ring with a halogen atom (3-fluoro-α-PHP and 4-fluoro-α-PHP), a methyl group (MPHP), or two methoxy groups (3,4-dimethoxy-α-PHP) or with the association of a halogen atom and a methyl group in the phenyl ring (MFPHP) [13,15,27,60,65,66,67,68,69], as can be seen in Figure 3.

### 3.3. Toxicokinetics

Alpha-PHP is known as a recreational drug of abuse and has no pharmacological therapeutic purpose. Thus, it can be abused through oral, nasal, sublingual, rectal, or intravenous application. The following table shows the relationship between the consumed dose and the proportion of the effect triggered through the various routes of administration (Table 1) [19].

In accordance with each route of administration, the latency and action time are different. Thus, in Table 2 is described the effect duration considering the various routes of α-PHP consumption [19].

Synthetic cathinones, more specifically, pyrovalerone, exert their psychostimulant effects, desired by the consumer, through the potent and selective inhibition of dopamine (DA) and norepinephrine (NE) neurotransmitter reuptake, since their transporters, DAT and NET, respectively, are inhibited [70,71,72]. Alpha-PHP mainly inhibits NET and DAT, but also has little impact on serotonin reuptake transporters (SERT), and do not enhance the release of neurotransmitters, unlike other synthetic cathinones [60,70,73,74,75]. Thus, α-PHP has moderate potency and high and low affinities for DAT and NET, respectively, and very low affinity and potency for SERT [19,73,76].

**Figure 4:**
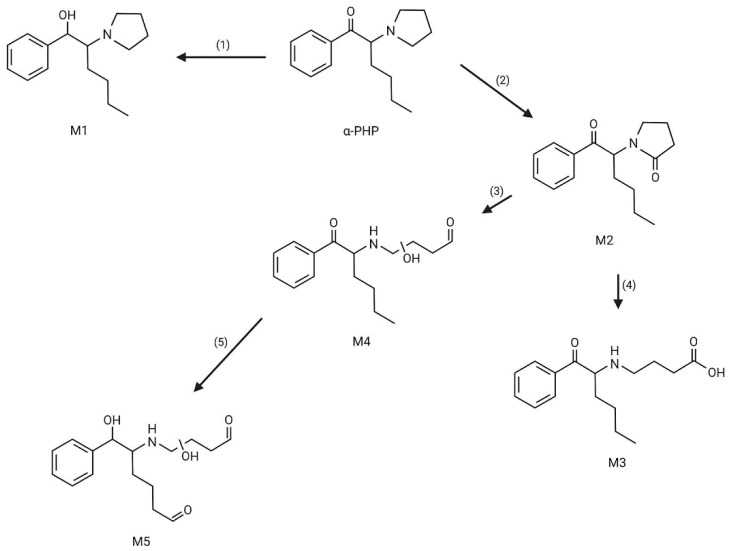
α-PHP metabolites: M1—alcohol dihydro-α-PHP; M2—2”-keto-oxidized pyrrolidone derivate; M3—4-(1-oxo-1-phenylhexan-2ylamino)butanoic acid; M4—n-hydroxy-4-(1-oxo-1-phenylhexan-2-ylamino)butanal; M5—6-hydroxy-5-(n-hydroxy-4-oxobutylamino)-6-phenylhexanal. (1) β-keto reduction; (2) hydrolysation and oxidation; (3) pyrrolidine ring cleavage and oxidation; (4) pyrrolidine ring cleavage and hydroxylation; (5) oxidation/reduction.

Alpha-PHP is mainly excreted in unaltered and in metabolized forms through urine. It has a half-life (T_1/2_) of 37 h and is eliminated from the human body in around 150 h, since more than 94% of the original consumed dose is removed after four half-lives T_1/2_ [77]. In fact, α-PHP is extensively metabolized and, to date, 19 phase I metabolites and 9 phase II metabolites have been identified [28]. It is mainly metabolized in the liver, and the most frequent phase I pathways are the reduction of the keto group; pyrrolidine ring didesalkylation; hydroxylation and oxidation of the aliphatic chain; pyrrolidine ring oxidation followed by cleavage of the ring; hydroxylation, reduction and oxidation; and a combination of these processes [78,79]. The major α-PHP metabolites are as follows: alcohol dihydro-α-PHP (M1: OH-α-PHP) produced through the β-keto reduction of α-PHP; the 2”-keto-oxidized pyrrolidone derivate (M2: 2”-oxo-α-PHP) produced through α-PHP hydrolysation and oxidation; pyrrolidine ring-cleaved products in aminobutyric acid and hydroxylated aldehyde forms produced through the oxidation and hydroxylation of 2”-oxo-PHP, respectively (M3: 4-(1-oxo-1-phenylhexan-2ylamino)butanoic acid; M4: n-hydroxy-4-(1-oxo-1-phenylhexan-2-ylamino)butanal); and the hydroxylated, β-keto-reduced alkyl chain and aminobutyl rest dialdehyde produced through the possible combined oxidation/reduction of other possible metabolites (M5: 6-hydroxy-5-(n-hydroxy-4-oxobutylamino)-6-phenylhexanal) [32,77,78,79]. Regarding phase II metabolization, glucuronide β-keto-reduced alcohols, including OH-α-PHP, are the most frequent [79]. All the metabolites described above are represented in Figure 4.

## 4. α-Pyrrolidinoisohexanophenone (α-PiHP)

### 4.1. Identification

Alpha-PiHP, as mentioned above, is a α-PHP isomer and therefore is also considered a pyrovalerone derivative from the class of synthetic cathinones [25]. Like α-PHP, this substance can be found in solid crystal and powder forms. Alpha-PiHP hydrochloride, in its pure form, tends to be odourless and can vary from off-white to white [25,34,80,81,82,83,84]. This substance has been sold on the streets under its original name and the same name as synthetic cathinones and has already been reported in a branded product called “Insomnia” [25]. In Portugal, α-PiHP is not known by a specific name, which is why it obtained a nickname that is a variant of “bloom”, the common name for α-PHP [57,59].

### 4.2. Chemistry

**Figure 5:**
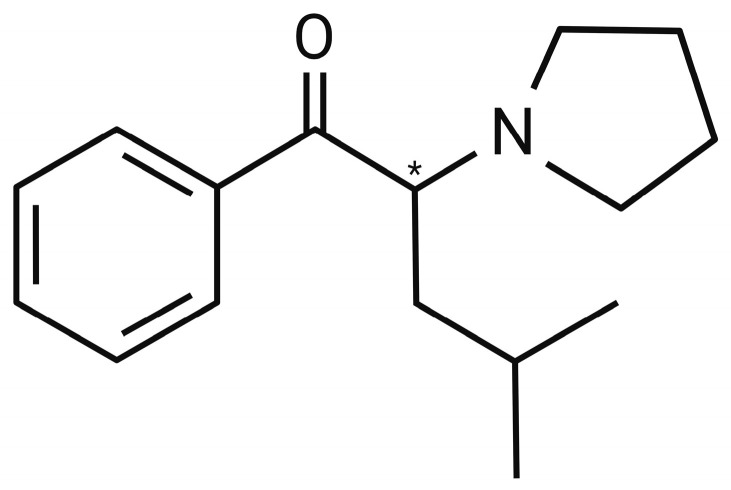
Chemical formula of α-pyrrolidinoisohexanophenone (α-PiHP).

Even though they are isomers, some of the chemical properties of α-PiHP differ from those of α-PHP. Alpha-PiHP’s IUPAC name is 4-Methyl-1-phenyl-2-(pyrrolidine-1-yl)pentan-1-one, and its molecular weight is the same as that of the isomer (245.36 g/mol). Structurally, it has the same composition as α-PHP (C_16_H_23_NO) but with the methyl group shifted from position 5 to 6 of the side chain, as shown in Figure 5. In addition, as well as its isomer, its molecule has a chiral centre (*) located in the α-carbon of the side chain, which makes it possible for it to form an enantiomeric pair ((*S*)-α-PiHP and (*R*)-α-PiHP), and even if it is more likely to appear as a racemic mixture, their individual emergence cannot be excluded [23,25].

### 4.3. Toxicokinetics

To date, α-PiHP is not known to have any therapeutic potential in medical use and has abuse liability and psychostimulant properties similar to other synthetic cathinones such as α-PHP [25]. It can be consumed through oral, intravenous, rectal, and nasal (insufflation and inhalation) routes. Table 3 compiles the range of doses and their proportional effects, and Table 4 their duration, both considering the different routes of administration [25,85].

Similarly to its isomer, α-PiHP also works by binding to monoamine transporters and inhibiting their reuptake, showing higher selectivity and potent reuptake inhibition for/in DAT and the NET compared to SERT. Thus, α-PiHP proves to be a potent DAT inhibitor due to its isomer α-PHP and other synthetic cathinones [25,86].

**Figure 6:**
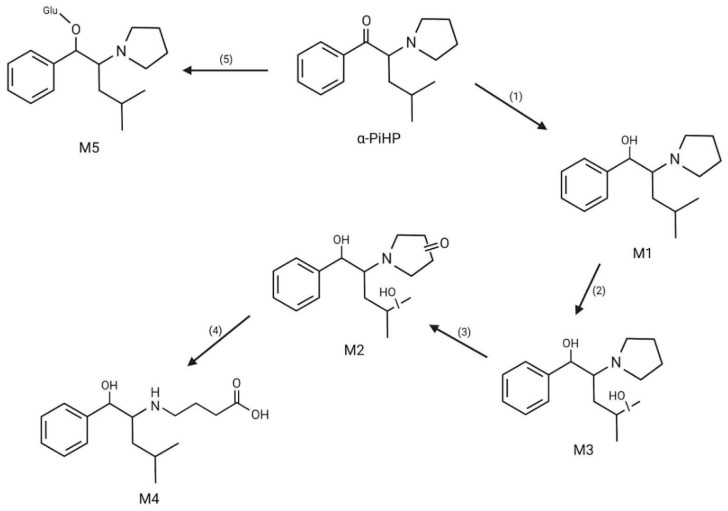
α-PiHP metabolites. (1) reduction of the β-keto group; (2) oxidation, hydroxylation, and reduction; (3) reduction and hydroxylation; (4) ring cleavage and carboxylation; (5) glucuronidation.

To date, the metabolism of αPiHP is not fully understood, so it is to be expected that it is related to its isomer, comprising the steps of β-keto group reduction and some oxidations and *N*-dealkylations [25]. In fact, eight phase I metabolites and two phase II metabolites have been identified so far. The most frequent metabolites found are those from phase I, the main two of which are formed by reduction of the β-keto group (M1) and by the combined oxidation of the pyrrolidine ring, aliphatic hydroxylation, and β-keto group reduction (M2). Three other metabolites, two phase I and one phase II, are also abundant and come from the combination of keto reduction and isoalkyl chain hydroxylation (M3), the cleavage of the ring and carboxylation (M4), and the glucuronidation of M1 (M5) [87]. All mentioned metabolites are included in Figure 6.

## 5. Adverse Effects

Pyrovalerone derivatives’ influence on DAT and NET and brain permeability propitiate higher sympathomimetic toxicity, and a higher DAT/SERT inhibition ratio indicates increased abuse potential and consumer addiction [60,86,88]. Furthermore, pyrovalerone is very likely to cross the blood–brain barrier easily due to its higher lipophilicity and documented high transmembrane permeability and potential active transport. Due to this fact and their potency in DAT and NET, their psychostimulant effects in humans can be explained at very low doses, being at least 10 times more potent DAT inhibitors than cocaine and methamphetamine [72,88]. In α-pyrrolidinophenones, the pyrrolidine ring and the α-carbon alkyl chain are determinants in DAT/NET activity, and the longer the chain, the greater the potency [89].

The most frequently reported signs and symptoms associated with the consumption of synthetic cathinones are cardiac, psychiatric, and neurological [8]. The cardiovascular effects include palpitation, tachycardia, and hypertension; the psychological effects are agitation, dysphoria, anxiety, panic, paranoia, hallucinations and depression; and the neurologic effects encompass aggressiveness, memory loss, headache, irreversible central nervous system damage, dizziness, tremors, and seizures. Moreover, there are also reported ophthalmologic effects, such as blurred vision and mydriasis, gastrointestinal symptoms like nausea, vomiting, and abdominal pain, and others such as fever and hyponatremia [8,28,90,91,92]. In particular, the use of α-PHP can lead to associated adverse effects such as agitation and compulsive behaviour, pronounced paranoia and anxiety, hallucinations, unconsciousness, hypertension, tachycardia and cardiac arrest, dehydration, nausea and vomiting, seizures, the suppression of physiological functions (e.g., urination), and rhabdomyolysis, and in some cases can cause death, as reported above [19,28,74,77]. When α-PHP’s action time has passed, the hangover of the consumer is characterized by a depressed mood, irritability, headache, and insomnia [19]. As is the case with its isomer, α-PiHP shows increased abuse potential due to its higher DAT/SERT inhibition ratio, and it also tends to be very toxic [25,86]. In terms of adverse effects, the ones that have been reported are mostly similar to α-PHP, such as psychiatric and neurological effects. Within these effects prevail paranoia, hallucinations, agitation, and unconsciousness. Moreover, cardiac effects can be present, such as tachycardia, vasoconstriction and cardiac arrest, muscular effects such as tremors and rhabdomyolysis, or even death, as in the fatal cases mentioned above [24,25,33,34,83]. Comparing the effects on behaviour of α-PiHP and α-PHP administration, it can be deduced that both have very similar locomotor stimulant and discriminative stimulus effects [89,93,94].

## 6. Forensic Toxicological Analysis

NPS detection in biological specimens remains a challenge for forensic and analytical toxicology due to the ease of market introduction and the speed at which they are synthesized and modified at [10,95]. Thus, there are a few published forensic studies dedicated to the analytical determination of α-PHP and α-PiHP substances. Table 5 summarizes all the published cases in the literature of forensic toxicology and their respective results for the detection and determination of those synthetic cathinones.

## 7. Conclusions

Alpha-PHP and -PiHP, as with many others synthetic cathinones, are currently not only drugs with a high power of abuse and addiction, but also substances with a very high rate of toxicity and mortality. This review aimed to bring together all the information on the two pyrrolidine derivates, α-PHP and α-PiHP, covering legislative, physicochemical, toxicological, and analytical aspects. Thus, it is possible to ascertain the dangerousness of both substances due to the adverse effects they can have on consumers, mainly at the cardiac, neurological, and psychological levels. Considering all the published cases, it can be concluded that most of these are fatal intoxications, and given the novelty of this type of substance, few cases have been shared with the scientific community.

In conclusion, NPSs’ ease of introduction on the market and their related dangers are both a massive issues in public health and challenges for forensic toxicologists, who are trying to respond to these types of substances by implementing methods to analyse them, although their identification is hindered by their ease of production and modification. This is clear when considering α-PHP and α-PiHP, as they are only identified as positional isomers because they both exert identical effects. In addition, the lack of effective legislation also favours the national and international dissemination of such substances. Therefore, further studies need to be carried out so that NPS data are always up to date, and more comprehensive legislation needs to be implemented for better control.

## Figures and Tables

**Table 1:** α-PHP dose ranges and the respective intensity of the effect produced through the various routes of consumption [19].

Effect Intensity	Oral (mg)	Nasal Sublingual Rectal (mg)	Intravenous Inhaled (mg)
First	3–5	2–4	1–2
Light	5–15	4–10	3–6
Normal	10–25	10–20	6–15
Strong	25–40	20–35	15–25
Very strong	40+	35+	25+

**Table 2:** α-PHP effect duration through the different routes of administration [19].

Effect Duration	Oral	Nasal Sublingual	Intravenous Inhaled
Latency time	15–45 min	5–15 min	A few seconds/ minutes
Main effect	4–6 h	2–4 h	30–90 min
Afterglow	4–10 h	4–10 h	2–5 h

**Table 3:** α-PiHP dose ranges and their effect intensity when administered through the various routes of consumption [85].

Effect Intensity	Oral (mg)	Nasal Insufflation (mg)	Nasal Inhalation (mg)
First	3–5	1–3	1–2
Light	5–15	3–10	2–10
Normal	10–30	10–25	10–20
Strong	30–50	25–40	20–30
Very strong	50+	40+	30+

**Table 4:** α-PHP effect duration through the different routes of administration [85].

Effect Duration	Oral	Nasal Insufflation	Nasal Inhalation
Latency time	30–60 min	1–2 min	1 min
Main effect	2–5 h	2–4 h	1–3 h
Afterglow	6–12 h	6–12 h	6–12 h

**Table 5:** α-PHP and -PiHP determination in biological specimens of reported forensic cases published in the literature.

Analytical Technique	Substance	Specimens	Concentration (ng/mL)	Specimen Preparation	Reference
LC-MS/MS	α-PiHP	Blood Urine Bile Bloody fluids Tissue homogenates	69 2072 341 33–448 7–478 ng/g	LLE	[24]
LC-MS/MS	α-PiHP	Femoral blood Heart blood Dural venous sinus blood Vitreous humour Cerebrospinal fluid Bile Solid tissues	2377 1133 966 1492 1350 540 9–2146 ng/g	LLE	[34]
GC-MS/MS	α-PiHP	Blood	611	LLE and derivatization	[96]
LC-MS/MS	α-PHP	Urine	32–5940	LLE	[78]
LC-MS/MS	α-PHP	Blood Urine	<1–12 136	LLE	[29]
LC-MS/MS	α-PHP	Hair	3600–4700 pg/mg	Washing with organic solvent and SPE	[97]
LC-MS/MS	α-PHP	Blood Urine Bile Solid tissues Hair	15.3 5.6 1.2 3.5–83.8 ng/g 1078 pg/mg	SPE	[28]
GC-MS-EI	α-PHP	Peripheral blood Cardiac blood	15–227 27-170	SPE	[3]
GC-MS-EI	α-PHP	Gastric contents Urine	INA	LLE	[26]
LC-MS/MS	α-PHP	Serum	175	QuEChERS	[77]
LC-MS/MS	α-PHP	Urine Serum	1–300 4–10	LLE	[27]
LC-HRMS	α-PHP	Blood Urine Hair	230 3430 19–100 pg/mg	SPE	[30]
LC-MS/MS	α-PHP	Blood Urine	10–153 10–2520	LLE	[31]
LC-MS/MS	α-PiHP	Blood Urine	5.0 722.2	INA	[33]
LC-MS/MS	α-PiHP	Blood Urine Vitreous humour Gastric contents Brain Liver	6.1 31.7 2.5 246 7.8 ng/g 0.0 ng/g	LLE	[83]

INA: information not available; LLE: liquid–liquid extraction; SPE: solid-phase extraction; QuEChERS: quick, easy, cheap, effective, rugged, and safe sample extraction.

## Data Availability

Data sharing is not applicable to this article.
